# Resource analysis and modifications of quantum computing with noisy qubits for elliptic curve discrete logarithms

**DOI:** 10.1038/s41598-024-54434-w

**Published:** 2024-02-16

**Authors:** Jinyoung Ha, Jonghyun Lee, Jun Heo

**Affiliations:** School of Electrical Engineering, Korea University, Seoul, 02841 Republic of Korea

**Keywords:** Quantum resource analysis, Quantum algorithm, Quantum error correcting code, Quantum physics, Electrical and electronic engineering, Computer science

## Abstract

We estimate the number of physical qubits and execution time by decomposing an implementation of Shor’s algorithm for elliptic curve discrete logarithms into universal gate units at the logical level when surface codes are used. We herein also present modified quantum circuits for elliptic curve discrete logarithms and compare our results with those of the original quantum circuit implementations at the physical level. Through the analysis, we show that the use of more logical qubits in quantum algorithms does not always lead to the use of more physical qubits. We assumed using rotated surface code and logical qubits with all-to-all connectivity. The number of physical qubits and execution time are expressed in terms of bit length, physical gate error rate, and probability of algorithm failure. In addition, we compare our results with the number of physical qubits and execution time of Shor’s factoring algorithm to assess the risk of attack by quantum computers in RSA and elliptic curve cryptography.

## Introduction

A variety of quantum computing techniques, ranging from algorithms to physical devices, have been actively investigated since Peter Shor proposed a polynomial-time quantum algorithm for finding discrete logarithms and factoring integers^1^. Several corporations, including Google and IBM, have pioneered efforts to make quantum computers feasible^2,3^.

However, the current level of quantum computers has clear limitations such as high gate error rates and small number of physical qubits. For this reason, research on noisy-intermediate-scale quantum operation is being actively carried out^4,5^, and research for performing basic quantum error-correcting (QEC) code in a quantum processor is also being conducted^6,7^. Quantum processor architecture research is also being performed to reduce the resources necessary for quantum computers^8–13^, as well as quantum computing software research for efficient quantum computer operation^14–22^.

Meanwhile, elliptic curves are utilized to create public key methods, such as key exchange^23^ and digital signatures^24,25^, which are widely employed in cryptographic systems. NIST curves P-256, P-384, and P-521, which are Weierstrass curves over special primes of sizes 256, 384, and 521 bits, respectively, are notable curves with widespread use. Elliptic curve cryptography is a public key cryptography approach based on the algebraic structure of elliptic curves over finite fields. The difficulty of computing discrete logarithms in elliptic curve groups, that is, the elliptic curve discrete logarithm problem, is used to secure elliptic curve cryptography.

The quantum resources requirements for Shor’s factoring algorithms have been investigated^26–31^. However, to the best of our knowledge, there are few studies on the physical resource analysis of Shor’s algorithm for elliptic curve discrete logarithms. Furthermore, as the outcomes of these assessments vary greatly, depending on the assumptions used, examining the resources required under various scenarios is vital.

**Figure 1:**
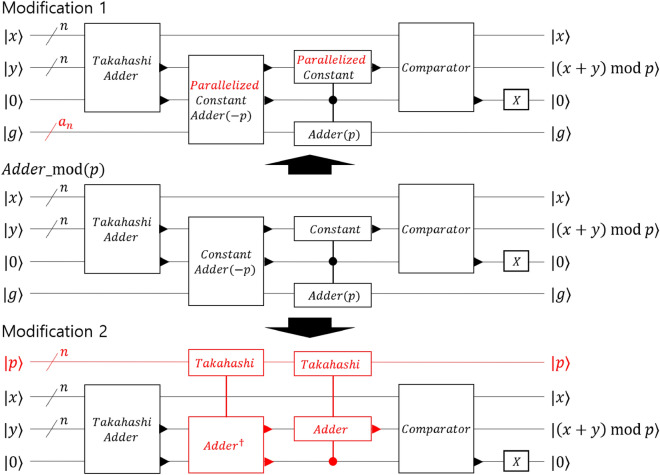
Slight modifications of modular operations in the Roetteler algorithm. The modified portions of the existing algorithm are marked in red. Modification 1 transforms the serial constant adders in the Roetteler algorithm into parallel constant adders^32^. Modification 2 changed the serial constant adders for modular operation in the Roetteler algorithm to the Takahashi adder^33^ by using more logical qubits for data.

Throughout the analysis, we adopt the resource analysis method presented in article^31^. In addition to the resource analysis, we modified the algorithm to reduce the required resources of the algorithm as illustrated in Fig. 1. We performed the modifications by focusing on the method of performing modular operations in the Roetteler algorithm (RA)^34^. First, we modify the serial constant adder to a parallel constant adder. As the parallel constant adder uses dirty ancilla qubits to reduce the operation depth^32^, the operation speed can be increased without using additional logical qubits for data. Second, we modified the constant adders to add or subtract *p* for modular operation into a Takahashi adder^33^. Takahashi adders need to use additional logical qubits for data because they need to input the number to be added as quantum values; however, as the structure is simple, it is expected to speed up the operation.

The contributions of this study are as follows. First, we express the number of physical qubits $N_{phy}$ and execution time $T_r$ required for RA as a closed-form equation for the bit length in elliptic curve cryptography. In addition, we proposed two types of modified algorithms to reduce the resources required for the RA: an algorithm that parallelizes a constant adder, and an algorithm that transforms constant adders for modular operation into Takahashi adder. We performed a resource analysis of the two modified algorithms; in the case of the first modified algorithm, the $N_{phy}$ is increased, but the execution time is reduced compared to the original RA. In the case of the second modified algorithm, the $N_{phy}$ remained almost unchanged, whereas the execution time was confirmed to be the shortest among the two modified algorithms and original RA. These results suggest that using the Takahashi adder can be more efficient than using the constant adder, even if additional logical qubits are used when performing modular operations. Finally, we compared the resources required for attacking elliptic curve cryptography with the resources required for attacking RSA analyzed in article^31^ and confirmed again that elliptic curve cryptography is more vulnerable to quantum computing attacks at the physical level.

## Related works

This section introduces Shor’s algorithm for elliptic curve discrete logarithms and review RA^34^. We then compare our work to a previous study that estimated the resources of RA.

### Shor’s algorithm for elliptic curve discrete logarithms

Several methods for implementing Shor’s algorithm for elliptic curve discrete logarithm have been developed since the proposal of Shor’s algorithm. Although previous studies implemented the algorithm in different ways, they all follow the general structure of Shor’s algorithm. Point addition performs the following operations:

1 $$\begin{aligned} \frac{1}{2^{n+1}} \sum _{k,l=0}^{2^{n+1}-1}|k,l\rangle \rightarrow \frac{1}{2^{n+1}}\sum _{k,l=0}^{2^{n+1}-1}|k,l\rangle |k\cdot Q+l\cdot P\rangle , \end{aligned}$$

where *P* and *Q* are the generator and public keys of elliptic curve cryptography, respectively. The Eq. (1) can be performed by implementing point addition on the elliptic curve as a quantum circuit. In 2003, Proos and Zalka^35^ demonstrated the implementation of Shor’s algorithm for elliptic curve discrete logarithms. They concluded with a table of resource estimations for the number of logical qubits and time based on the bit length of the elliptic curve at a logical level. Based on this work, In 2017, Roetteler et al.^34^ developed quantum circuit for elliptic curve discrete logarithms, and then used this concrete implementation to automatically compute resource estimates at a logical level.

### Previous works

We review several studies analyzing the resources of physical quantum computing using Shor’s algorithm for discrete logarithms. We summarized previous works and our work in Table 1. Roetteler *et* *al*.^34^ presented precise resource estimates for quantum circuits that implemented Shor’s algorithm for elliptic curve discrete logarithm problem at a logical level. For example, they observed that Shor’s algorithm for 224-bit discrete logarithms required 2042 logical qubits and $7.73\times 10^{10}$ logical Toffoli depth. They also compared the quantum resources for solving the elliptic curve discrete logarithm problem to those required in Shor’s factoring algorithm, which were obtained in a recent study^32^. Their logical-level comparison indicated that the amount of qubits required to attack elliptic curves is fewer than that required to attack RSA for current settings at comparable classical security levels, implying that elliptic curve encryption is really an easier target than RSA. However, only the logical level was considered for their result, so their results did not consider various conditions such as $\epsilon _p$ and algorithm failure probabilities to be considered when the quantum circuit is applied to noisy quantum computers. In addition, as quantum algorithms that attack ciphers must be implemented by physical quantum computers, physical-level resource comparisons are required to ensure the risks of quantum computing attacks to elliptic curve cryptography and RSA.

Gheorghiu *et* *al*.^30^ analyzed the resources required by various algorithms, including RA for discrete logarithms, attacking the current cryptosystem at the physical level. They also compared the quantum vulnerability of RSA and elliptic curve cryptography for a fixed classical bit length at the physical level. They utilized a surface code that required fewer physical qubits to construct a logical qubit^36^, which is similar to our work. They performed resource analysis with $10^{-3}$ and $10^{-5}$ of $\epsilon _p$, and the relation between the required time and the $N_{phy}$ of the algorithm was expressed in a closed form by adjusting the level of parallelization for the magic-state factory. However, their closed formula does not contain parameters such as bit length, physical error rate, or algorithm failure probability; therefore, various situations cannot be considered.

**Table 1:** Summary of previous works and our work.

	Physical-level analysis	Magic-state factory parallelization	Closed-form formula
Roetteler^34^	X	X	X
Gheorghiu^30^	O	O	$\triangle$
Our work	O	X	O

“$\triangle$” indicates that the corresponding component has been partially considered. Gheorghiu’s closed-form formula is an expression of the $N_{phy}$ according to the time required when the bit length and physical error rate are determined, whereas our work derives the number of qubits and the time required using the bit length, physical error rate, and algorithm failure probability as parameters. Roetteler analyzed the required resources of the algorithm only at the logical level. As the magic-state factory is not used at the logical level, Roetteler’s work does not include magic-state factory parallelization. Our work does not consider magic-state factory parallelization because it assumes that the state stored in the magic-state factory cannot be stored for a long time and should be used immediately.

We utilized a rotated planar surface code that requires fewer physical qubits than than the method of article^37^. We assumed all-to-all connectivity between logical qubits. We utilized the MSD protocol described in article^8^. We also consider RA for discrete logarithms. In contrast to prior studies, our study describes the $N_{phy}$ and the required time as a closed-form formula. Our closed-form formula considers bit length, physical gate error rate, and algorithm failure probability; therefore, obtaining the resources required in various situations is easy. Unlike article^30^, we did not consider magic-state factory parallelization because we assumed that states made in the magic-state factory should be used immediately. In addition, we compared the required resources for the algorithms for discrete logarithms and factoring under the same conditions.

## Methods

### Resource analysis scheme

As the current level of physical quantum gates has a very high error rate, techniques such as QEC code and MSD should be used to create logical qubits and logical operations with low error rates. We performed a resource analysis using the physical-level analysis method and the equation for the required resources used in article^31^. In this section, we briefly introduce the techniques used for this physical-level analysis and introduce a closed-form formula for $N_{phy}$ and $T_r$.

As mentioned above, we used QEC code to generate a logical qubit with a low gate error rate. We selected a rotated planar surface code from a variety of QEC codes^36^. The number of physical qubits required to construct one logical qubit is $2d^2-1$ when the code distance is *d*. The following relationship is established between the *d*, $\epsilon _p$, and logical gate error rate $\epsilon _L$ required by the algorithm in the rotated surface code^8^.

2 $$\begin{aligned} d=2\cdot \lceil \frac{\log (10\epsilon _{L})}{\log (100\epsilon _p)}\rceil -1. \end{aligned}$$

In Eq. (2), $\lceil x \rceil$ is the function that takes real number *x* as an input and returns the least integer greater than or equal to *x*. Therefore, we can determine the distance of the surface code using KQ formalism to obtain the $\epsilon _L$ required by the algorithm.

To perform the *T* gate with a low error rate in QEC codes, the $|A\rangle = |0\rangle +\exp (i\cdot \pi /4)|1\rangle$ state with a low error rate is required. MSD uses multiple noisy $|A\rangle$ states and outputs a smaller number of more reliable $|A\rangle$ states.

**Table 2:** Summary of $T_m$ and $t_T$ according to the distillation level.

Distillation level	$T_m$	$t_T$
1	$T_{m,1}=32\cdot (2d_1^2-1)$	$6.5\cdot d_1\cdot c_t$
2	$T_{m,2}=32\cdot (2d_2^2-1)+8\cdot T_{m,1}$	$6.5\cdot \max (2d_1,d_2)\cdot c_t$
3	$T_{m,3}=32\cdot (2d_3^2-1)+8\cdot T_{m,2}$	$6.5\cdot \max (4d_1,2d_2, d_3)\cdot c_t$

$T_m$ and $t_T$ are determined by the code distances $d_1$, $d_2$, and $d_3$ used for the MSD.

As in article^31^, we used Fowler and Gidney’s MSD protocol^8^. We performed various levels of MSD based on the $\epsilon _L$ required by the algorithm. The code distances for MSD $d_1$, $d_2$, and $d_3$ all have odd values greater than or equal to 15. As shown in Table 2, the number of physical qubits required to make one *T* gate, $T_m$, and the time required for MSD, $t_T$, are expressed as functions of $d_1$, $d_2$, and $d_3$. In Table 2, $c_t$ denotes the cycle time of the surface code^8^. Many studies have assumed that $c_t=200$ ns^26,30^, and we have adopted that assumption. We set $d_1$, $d_2$, and $d_3$ such that $T_m$ is as minimal as possible while still satisfying the $\epsilon _L$ requirement. As the $T_m$ increased dramatically when the distillation level increased, we adjusted the code distances to perform distillation at the lowest possible level.

As shown in article^31^, the $N_{phy}$ and the $T_r$ can be expressed as

3 $$\begin{aligned} N_{phy}&=(2\cdot (2\lceil \frac{\log (\frac{10 p_{fail}}{KQ})}{\log (100\epsilon _p)}\rceil -1)^2-1)\cdot (K+N_{CNOT})+N_T\cdot T_m, \end{aligned}$$

4 $$\begin{aligned} T_r&=\frac{D\cdot t_T}{1-p_{fail}}. \qquad \qquad \qquad \qquad \qquad \qquad \qquad \qquad \qquad \quad \end{aligned}$$

In Eq. (3), *K* is the number of logical qubits required by the algorithm, $N_{CNOT}$ is the maximum number of concurrent *CNOT* gates, and $N_T$ is the maximum number of concurrent *T* gates. In Eq. (4), *D* is the *T* depth of the algorithm. In Eqs. (3) and (4), $p_{fail}$ is the probability of the failure of the algorithm. When elliptic curve logarithms on an elliptic curve is defined over *n*-bit prime field, the RA has $K=9n+2\lceil \log _2 (n) \rceil +10$.

### Decomposition of algorithm at the logical level

Similar to the analysis used in article^31^, the algorithm is decomposed into *X*, *Z*, *CNOT*, and *T* gates, and the $\epsilon _L$ for the algorithm to operate properly is obtained using KQ formalism. Using this logical gate error rate obtained through KQ formalism, we can determine the distance of the surface code and the level and distance of the MSD. In addition, we obtained the *T* depth to identify the $T_r$. To determine the number of magic-state factories to be prepared, we counted $N_T$. Considering the *CNOT* operation using the lattice surgery method^36^, we also determined that the $N_{CNOT}$.

KQ formalism is described as follows^38^:

5 $$\begin{aligned} p_{fail}=KQ\epsilon _L. \end{aligned}$$

In Eq. (5), $p_{fail}$ is the algorithm failure probability. We can determine $\epsilon _{L}$ by obtaining *K* and *Q*. Previous studies have performed analyses using $p_{fail}$ as a fixed value^27,28^. On the contrary, we set $p_{fail}$ as a variable to confirm the change in the required resources of the algorithm according to the change in $p_{fail}$.

Because the values of *K* and *Q* depend on the algorithm, we decomposed the RA to obtain *K* and *Q*. First, we confirmed that decomposing the Toffoli gate using the method presented in article^39^ would result in Q of 11 and T depth of 3. The majority of the operations in the RA are modular operations that require a constant adder gate. In 2016, Häner *et* *al*. developed a quantum constant adder using a divide-and-conquer method^32^. Häner’s constant adder includes serial and parallel versions, and the RA uses a serial version of a constant adder. The elementary gate step $Q_{Roe}$ and *T* depth $D_{Roe}$ of the RA are expressed as follows (Please see supplementary information).

6 $$\begin{aligned} Q_{Roe}&=2472n^3\log _2 n+10316n^3+1442n^2\log _2 n-2222n^2,\end{aligned}$$

7 $$\begin{aligned} D_{Roe}&=576n^3\log _2 n+2796n^3+336n^2\log _2 n-510n^2. \end{aligned}$$

As the *CNOT* gates are serially executed in the Takahashi adder, there are no cases where two or more *CNOT*s are executed simultaneously. Thus, $N_{CNOT}=1$. In addition, because three *T* gates are used simultaneously in the Toffoli gate, and because there are no cases where the Toffoli gate is used simultaneously in the entire algorithm, the maximum number of *T* gates used simultaneously throughout the algorithm is $N_T=3$.

### Slight modifications to the Roetteler algorithm

In this section, we modify the execution method of the constant adder in the RA and evaluate the change in the elementary gate step and *T* depth. As a constant adder is used throughout the RA, a little change in the constant adder causes a significant results. First, to reduce the required time for the algorithm, we replace Häner’s serial version constant adder with a parallel one. As the parallel constant adder performs operations simultaneously, the elementary gate step and *T* depth can be reduced, but the $N_{CNOT}$ and $N_T$ increases. Second, we modify the constant adder that adds or subtracts a modular number *p* to a Takahashi adder by additionally using *n* logical qubits. These additional logical qubits are used to store modular number *p* as a quantum state. We do not change all constant adders used in the RA but change only the constant adders that add or subtract the modular number *p* to Takahashi adders. As the Takahashi adder has fewer elementary gate steps than the constant adder, it also shortens the required time.

#### Constant adder parallelization

Häner *et* *al*. also presented a method to parallelize the constant adder by additionally using dirty ancilla qubits in article^32^. Because this method additionally uses dirty ancilla qubits, it has the advantage of not having additional logical data qubits used for parallelization while reducing the elementary gate step and *T* depth. However, when the constant adder is parallelized, $N_{CNOT}$ and $N_T$ increase, and additional logical qubits for performing *CNOT* gate and magic-state factories are required. We used parallel constant adder to reduce the time required for the algorithm, even if we risk an increase in the number of physical qubits. Let us define the number of Toffoli gates that are used simultaneously as $a_n$ and the number of *CNOT* gates that are used simultaneously as $b_n$ when performing an *n*-bit quantum constant adder. As the constant adder has the form of divide-and-conquer, the *n*-bit constant adder is expressed as the sum of the constant adders of the upper and lower half bits. Therefore, $a_n$ and $b_n$ follow the recurrence relation

8 $$\begin{aligned} a_{n}=a_{\lceil \frac{n}{2} \rceil }+a_{\lfloor \frac{n}{2}\rfloor },~ b_{n}=b_{\lceil \frac{n}{2} \rceil }+b_{\lfloor \frac{n}{2}\rfloor }, \end{aligned}$$

where the natural number $n\ge 2$ and $a_1=0, a_2=0,a_3=1, a_4=1$ and $b_1=0, b_2=1$. Although $a_n$ and $b_n$ cannot be expressed in a closed form, $a_n$ and $b_n$ for any *n* can be obtained using the values of $a_1, a_2,a_3, a_4$, and $b_1, b_2$. For example, when $n=13$, $a_{13}=a_7+a_6=(a_4+a_3)+(a_3+a_3)=4$ and $b_{13}=b_7+b_6=b_4+3b_3=(b_2+b_2)+3(b_2+b_1)=5$. Using $a_n$ and $b_n$, when the bit length is *n*, $N_{CNOT}$ and $N_T$ can be expressed as

9 $$\begin{aligned} N_{CNOT}=b_n,~ N_{T}=3a_n. \end{aligned}$$

Using this modification, we can redefine the elementary gate step and depth of the parallelized constant adder RA. The elementary gate step $Q_{Roe,parallel}$ and *T* depth $D_{Roe,parallel}$ of the parallelized constant adder RA are expressed as follows (please see supplementary information).

10 $$\begin{aligned} Q_{Roe,parallel}&=19796n^3+3422n^2, \end{aligned}$$

11 $$\begin{aligned} D_{Roe,parallel}&=5100n^3+834n^2. \end{aligned}$$

#### Using Takahashi adder instead of constant adder

Although the *n*-bit constant adder uses only *n* logical qubits and thus requires fewer qubits than adder, which requires additional logical qubits, the elementary gate step and *T* depth of the constant adder are larger than those of the adder. For example, the Takahashi adder requires elementary gate step of 27*n* and *T* depth of 6*n* approximately, assuming CNOT gate serialization. Similarly, ripple-carry adder of Cuccaro et al.^40^, knwon as CDKM adder, requires an elementary gate step of 25*n* and a *T* depth of 6*n* approximately, assuming CNOT gate serialization again. Unlike the Takahashi adder, the CDKM adder requires one additional logical ancilla qubit. The Draper adder^41^ requires $n+1$ steps of controlled rotation gate. However, The Draper adder requires approximate quantum Fourier transform(AQFT) before and after the operation and therefore requires an additional gate depth of $O(n\log n)$. Furthermore, we have to approximate the rotation gate as *H*, *S*, and *T* gates using the Gridsynth algorithm^42^. For example, when the degree of approximation is $10^{-10}$, a total of 253 *H*, *S*, and *T* gates are required and 102 *T* gates are required^42^. Therefore, Draper adder including AQFT was not considered in this paper because both the number of qubits used and the depth were larger than constant adder. Comparing the Takahashi adder and the CDKM adder, the CDKM adder has a slight advantage in terms of elementary gate steps compared to the Takahashi adder, but the *T* depth, which greatly affects the time required, is the same as Takahashi, and it uses one more logical qubit. In addition, when multiple-controlled adders are needed, such as modular inversion, Takahashi adders are more advantageous than CDKM adders. Therefore, when performing resource analysis, when comparing the method of replacing the constant adder with Takahashi adder and the method of replacing it with CDKM adder, the method using Takahashi adder uses slightly fewer physical qubits and takes the same amount of time (please see supplementary information). Therefore, we replaced constant adder with Takahashi adder. The disadvantage of the Takahashi adder is that the number to be added must be prepared in the quantum state. Therefore, we replaced only the constant adder for adding or subtracting the modular number *p*, which is frequently used in modular operations, with the Takahashi adder. Using this method, only *n* additional logical qubits must be generated to store modular number *p*.

As in the RA, it is assumed that the *CNOT* gates are serially performed on Takahashi adder to minimize $N_{CNOT}$. Therefore, as in the RA, $N_{CNOT}=1$ and $N_T=3$.

The elementary gate step $Q_{Roe,T}$ and *T* depth $D_{Roe,T}$ of the Takahashi adder version RA are expressed as follows (please see supplementary information).

12 $$\begin{aligned} Q_{Roe,T}&=15776^3 + 824n^2\log _2 n -804n^2,\end{aligned}$$

13 $$\begin{aligned} D_{Roe,T}&=4056n^3 + 192n^2\log _2 n -180n^2. \end{aligned}$$

## Results

**Table 3:** Comparing our estimation of $N_{phy}$ for the RA with^30^ based on our resource analysis results of required time.

bit length	160	192	224	256	384	521
Required time (s)	$1.40\times 10^6$	$2.47\times 10^6$	$3.99\times 10^6$	$5.37\times 10^6$	$2.25\times 10^7$	$6.09\times 10^7$
Number of qubits (proposal)	$3.52\times 10^6$	$4.08\times 10^6$	$4.63\times 10^6$	$5.81\times 10^6$	$8.32\times 10^6$	$1.23\times 10^7$
Number of qubits^30^	$3.92\times 10^6$	$5.73\times 10^6$	$5.95\times 10^6$	$5.87\times 10^6$	$8.60\times 10^6$	$1.92\times 10^6$

Table 3 shows the $N_{phy}$ in our study and in article^30^ with the several fixed required time. We assumed $\epsilon _p =10^{-3}$, $c_t=200$ ns, and $p_{fail}=0.01$. In article^30^, the required resources are expressed as a function of time and the $N_{phy}$. Therefore, the $N_{phy}$ estimation of ours and article^30^ are compared based on the required time for our analysis. As shown in Table 3, although our analysis uses a slightly fewer $N_{phy}$, they require similar resources.

**Figure 2:**
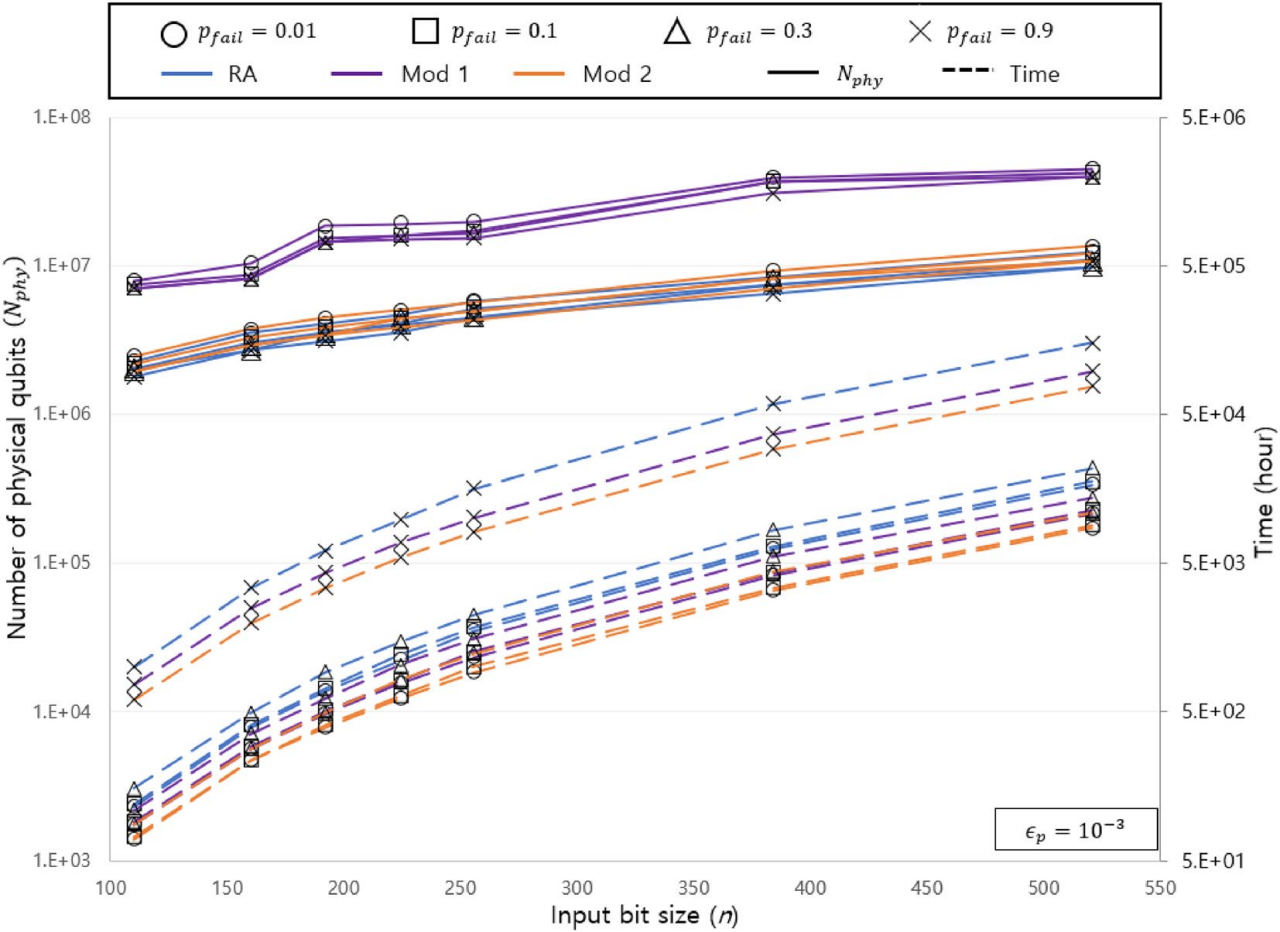
$N_{phy}$ and required time with different bit length. The solid line indicates the $N_{phy}$ and the dotted line indicates the required time. A circle represents $p_{fail}=0.01$, a square represents $p_{fail}=0.1$, a triangle represents $p_{fail}=0.3$, and an X represents $p_{fail}=0.9$.

Figure 2 shows the $N_{phy}$ and the required time of the RA and our modifications for $\epsilon _p$ of $10^{-3}$ and $p_{fail}$ values of 0.01, 0.1, 0.3, and 0.9 with various bit lengths *n*. Let us name the algorithm that parallelizes the constant adder in the RA as Mod 1, and the algorithm that changes the constant adder to Takahashi adder in the RA as Mod 2. For most bit lengths, the RA has about 0.9 times fewer $N_{phy}$ than the Mod 2, but in a specific bit length, the Mod 2 uses fewer qubits than the RA. For example, when $n=256$ and $p_{fail}=0.01$, the $N_{phy}$ of the RA is $5.81\times 10^6$, and the $N_{phy}$ of the Mod 2 is $5.68\times 10^6$. This point appears because the decrease in *Q* is larger than the increase in *K* due to the change in the algorithm, so the $\epsilon _L$ required by the algorithm increases. For example, when $n=256$ and $p_{fail}=0.01$, $\epsilon _L=8.49\times 10^{-18}$ of the RA and $\epsilon _L=1.46\times 10^{-17}$ of the Mod 2. This difference leads the code distances to change from $d=33, d_1=17, d_2=39$ in the RA to $d=31, d_1=17, d_2=37$ in the Mod 2, and these changes leads the Mod 2 to smaller $N_{phy}$. This suggests that even if more logical qubits are used, the $N_{phy}$ can be reduced if the elementary gate step of the algorithm is reduced.

When $p_{fail}=0.01$, the required time of the RA takes approximately 1.65 times longer than that of the Mod 2 when $n=110$, and the difference widens as *n* increases, showing a difference of approximately 1.97 times when $n=521$. This is because the order of the *T* depth of the RA is $O(n^3 \log n)$, whereas the order of the *T* depth of Mod 2 is $O(n^3)$.

Overall, the Mod 1 had a greater $N_{phy}$ than the Roetteler and Mod 2. The reason for this is that a large number of magic-state factories must be used simultaneously in the process of executing the Mod 1. The RA, for example, requires approximately 4 million physical qubits for data and approximately 0.7 million physical qubits for MSD when $n=224$ and $p_{fail}=0.01$, whereas the Mod 1 requires approximately 4.1 million physical qubits for data and approximately 15 million physical qubits for MSD. The Mod 1 requires a similar number of physical qubits for data as required by the RA, whereas it requires approximately 21 times the number of physical qubits for MSD.

Similar to the Mod 2, the parallelized algorithm has a *T* depth of $O(n^3)$; therefore, the required time is smaller than that of the RA. However, the Mod 1 takes longer than the Mod 2 because $D_{Roe,parallel}$ is larger than $D_{Roe,T}$.

**Figure 3:**
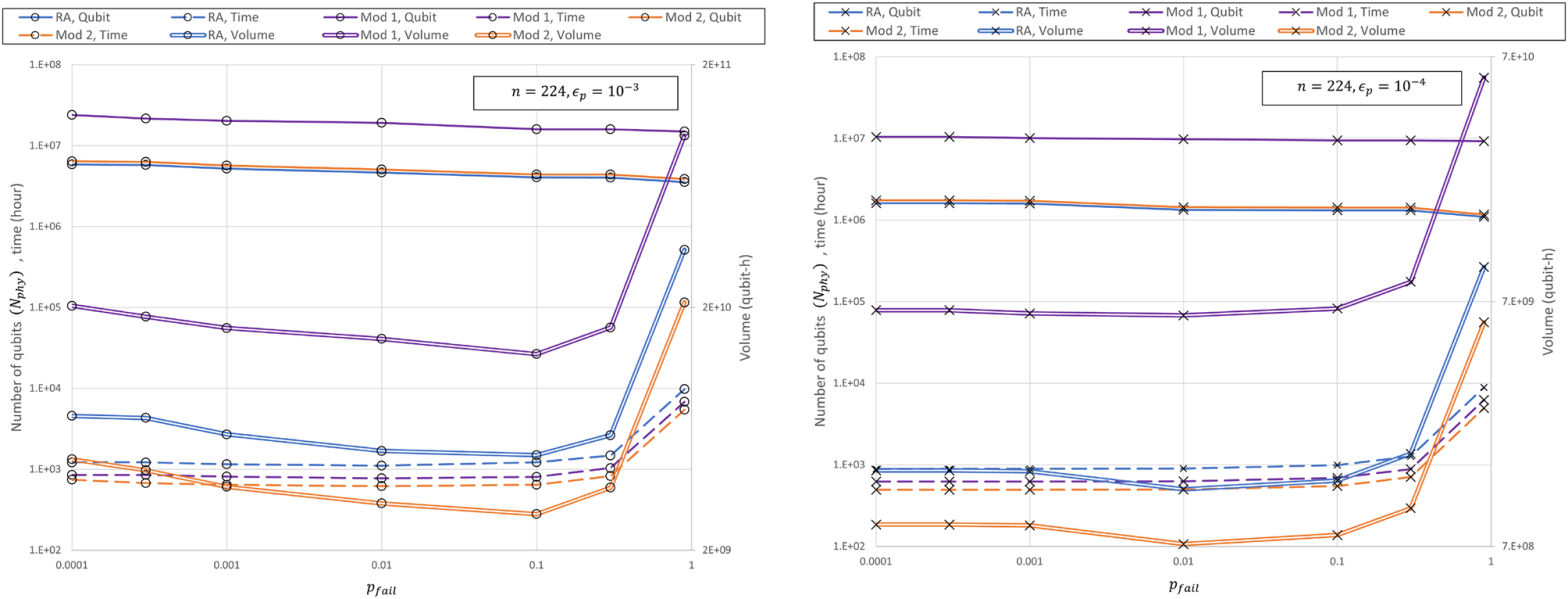
Quantum volume, $N_{phy}$, and required time for Roetteler, Mod 1, and Mod 2 for various $p_{fail}$s when (**a**) $\epsilon _p=10^{-3}$ and (**b**) $\epsilon _p=10^{-4}$. The solid line indicates the $N_{phy}$, the dotted line indicates the required time, and the double line indicates the required time. As in Fig. 2, RA is shown in blue, Mod 2 in purple, and Mod 2 in orange. In (**a**), all three algorithms have a minimum quantum volume when $p_{fail}=0.1$, and in (**b**), when $p_{fail}=0.01$, all three algorithms have a minimum quantum volume.

Figure 3 shows the quantum volume, $N_{phy}$, and $T_r$ for the Roetteler, parallel, and Mod 2 when the $\epsilon _p$ is $\epsilon _p=10^{-3}$ for Fig. 3a, and $\epsilon _p=10^{-4}$ in Fig. 3b, and $n=224$ for various values of $p_{fail}$s. The quantum volume is a product of the $N_{phy}$ and the $T_r$, and represents the overall complexity of the algorithm. Consistent with the result of article^31^, the quantum volume decreases as $p_{fail}$ decreases and then tends to increase again at some point. In Fig. 3a, the quantum volume shows the smallest values for all three algorithm when $p_{fail}$ approaches 0.1, whereas the quantum volume of all algorithms is the smallest when $p_{fail}$ is near 0.01, as shown in Fig. 3b. Consequently, together with article^31^, selecting an appropriate $p_{fail}$ value is required to conduct an effective algorithm, and the optimal $p_{fail}$ value fluctuates based on conditions such as the $\epsilon _p$ and *n*. This fact is expected to be applicable regardless of the algorithm.

In Fig. 3, the quantum volume of the Mod 1 is the largest, and the quantum volume of the Mod 2 is the smallest, regardless of $p_{fail}$ and $\epsilon _p$. Although the Mod 1 has higher volume than the RA, it may operate more efficiently in limited situations such as those where the physical qubits are sufficiently given and the algorithm execution time needs to be reduced. Overall, the quantum volume is the smallest among the three algorithms. Thus, the Mod 2 is generally the most efficient algorithm among the three algorithms. The difference between the Mod 2 and the other two algorithms is that the Takahashi adder is used instead of the constant adder for modular operations. Therefore, even if more logical qubits are used, it may be more efficient to use the Takahashi adder than the constant adder when performing modular operations.

**Figure 4:**
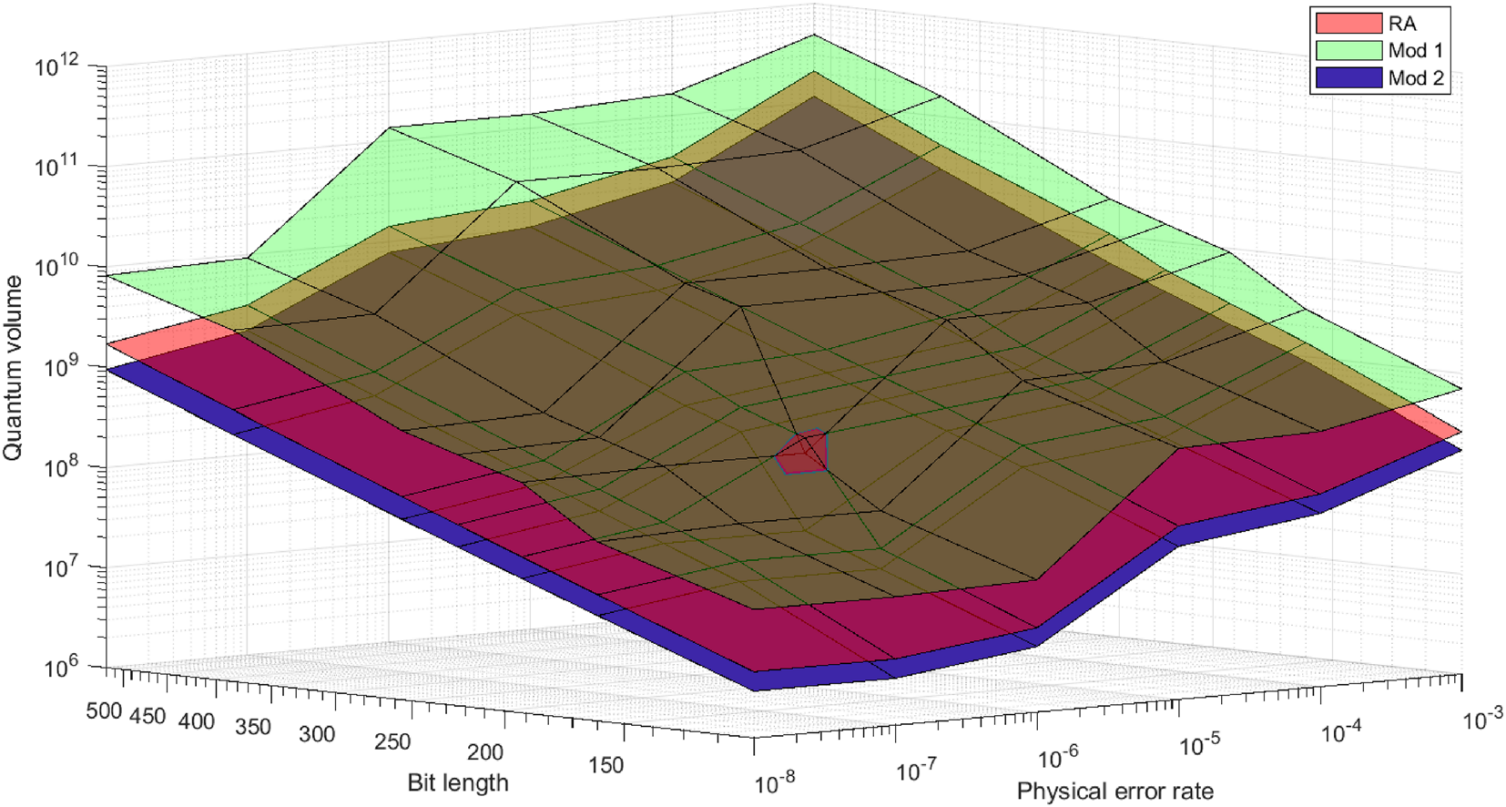
Quantum volume of the RA, Mod 1, and Mod 2 for various bit lengths and physical gate error rates. The red, green, and blue surfaces represent RA, Mod 1, and Mod 2, respectively.

Figure 4 shows the quantum volume of the RA and our modifications for different bit lengths and $\epsilon _p$s when $p_{fail}=0.01$. The point that increases rapidly in quantum volume is where the MSD level increases. Although the Mod 1 has a larger quantum volume than the RA in almost all situations, the Mod 1 has a smaller quantum volume than that of the RA When $\epsilon _p=10^{-6}$ and $n=192$. This is because the Mod 1 has a smaller *T* depth than the RA, so the the $\epsilon _L$ required by the Mod 1 is larger than that of the RA and the MSD level of Mod 1 is lower. As the $\epsilon _L$ required by the algorithm determines the MSD level, a corresponding situation may occur in some points. Therefore, the level of MSD is an important factor in determining the complexity of the algorithm.

**Figure 5:**
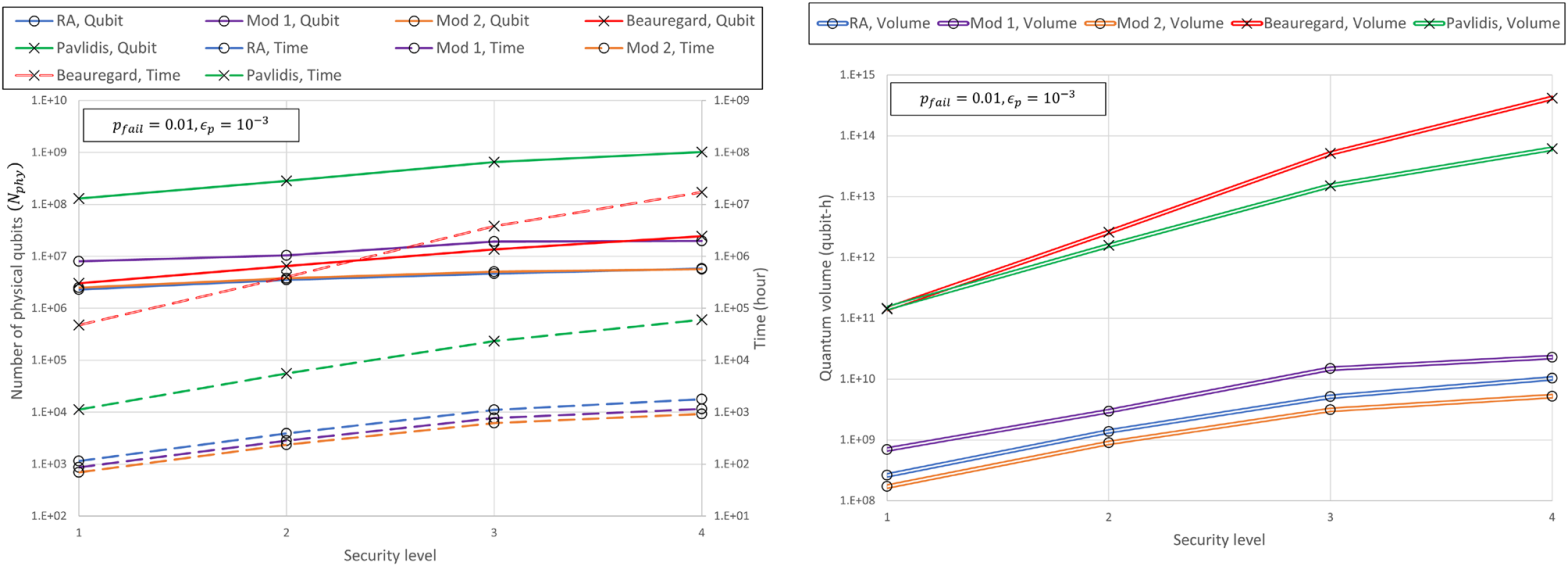
Comparison of (**a**) $N_{phy}$ and required time and (**b**) quantum volume for algorithms performing prime factorization and algorithms performing discrete logarithm when $p_{fail}=0.01$ and $\epsilon _p=10^{-3}$. The Beauregard algorithm for prime factorization is expressed as “Beauregard”, Pavlidis algorithm for factorization as “Pavlidis”. RA is expressed in blue, parallel in purple, adder in orange, Beauregard in red, and Pavlidis in green. The circle mark represents the algorithm for the discrete logarithm, and the X-mark represents the algorithm for prime factorization.

Figure 5 shows the quantum volume of our results and that of the two algorithms for factoring presented in article^31^ when $p_{fail}=0.01$, $\epsilon _p=10^{-3}$, and $c_t=200$ ns. The *x* axis represents the same level of security based on classical computing. Security levels 1, 2, 3, and 4 represent the discrete logarithm with bit lengths $n=110, 160, 224, 256$ and prime factorization with bit lengths $n=512, 1024, 2048$, and 3072, respectively. Overall, the factoring algorithm tended to be larger than the algorithms for discrete logarithms in terms of the required time, $N_{phy}$, and quantum volume. In addition, the increase in the required resources of the factoring algorithms according to the increase in the security level is larger than that of the discrete logarithm algorithm. In security level 1, the Beauregard algorithm has a $N_{phy}$ similar to the Mod 2 and the RA, whereas in security level 4, it uses slightly more qubits than the Mod 1. The quantum volume of the algorithm for discrete logarithms is much smaller than that for factoring, and the gap increases as the level of security in classical computing increases. For example, even at security level 1, the quantum volume difference between the Mod 2 and Beauregard algorithm is approximately $10^3$, and when going to the fourth level of security, the difference increases to $10^5$. Therefore, the elliptic curve cryptography is more vulnerable to the attack of quantum computing than the RSA, and the degree of this vulnerability is very large at the physical level.

## Discussion

We formulated closed-form equations for the number of physical qubits and execution time required by the Roetteler algorithm, assuming all-to-all connectivity, and compared the resources required for different bit lengths, physical gate error rates, and the probability of algorithm failure. We adopted the method of article^31^ to obtain closed-form equations of the required resource. In addition, we slightly modified the modular operation of the Roetteler algorithm. First, we parallelize the constant adder used for modular operations. Second, we replaced the constant adder used in the modular operation with the Takahashi adder using additional logical qubits. We analyzed the required resources of the modified algorithm and compared them with those of the Roetteler algorithm. The results show that among the three algorithms for discrete logarithms, the algorithm using the Takahashi adder is the most efficient. This result suggests that it is more efficient to use the Takahashi adder than using the constant adder when performing modular operations, even if logical qubits are additionally used. Through the fact that the Mod 2 uses fewer physical qubits than the Roetteler algorithm in a specific case, we confirmed that an increase in the number of logical qubits does not necessarily lead to an increase in the $N_{phy}$. In addition, considering that the Mod 1 with a large number of magic-state factories has more quantum volume and $N_{phy}$ than the Roetteler algorithm, we confirmed that reducing the number of magic-state factories to reduce the required resources is necessary. Finally, we compared the results of our analysis with those of article^31^, who analyzed the required resources of the algorithms for factoring. The analysis results show that in most cases, algorithms for discrete logarithms for quantum volume, $N_{phy}$, and execution time are smaller than those for factoring, and the higher the security level, the greater is the difference. In particular, we confirmed that the quantum volume of the algorithm for the discrete logarithm was smaller than the quantum volume of the algorithm for factoring by $10^3$ to $10^5$ times according to the security level. Therefore, elliptic curve cryptography is more likely to be attacked by quantum computing than RSA, and we confirmed that the degree is very large when compared at the physical level.

### Supplementary Information

Supplementary Information.

## Data Availability

The datasets used and/or analysed during the current study available from the corresponding author on reasonable request.
