# Supplementary material for: Resource analysis and modifications of quantum computing with noisy qubits for elliptic curve discrete logarithms
Source: Sci Rep. 2024 Feb 16;14:3927. doi: 10.1038/s41598-024-54434-w (PMC10873342; doi:10.1038/s41598-024-54434-w)
Supplement: Supplementary file 1 — Supplementary Information. [file 41598_2024_54434_MOESM1_ESM.pdf]

Supplementary Information For "Resource analysis and modifications of quantum computing with noisy qubits for elliptic curve discrete logarithms"

Jinyoung Ha[1], Jonghyun Lee[1], and Jun Heo[1,*]

[1]School of Electrical Engineering, Korea University, Seoul, 02841, Republic of Korea
[*]junheo@korea.ac.kr

ABSTRACT

Supplementary Information For "Resource analysis and modifications of quantum computing with noisy qubits for elliptic curve discrete logarithms"

Decomposition of Roetteler algorithm (RA) at the logical level

We will show how the elementary step gate step Q_{Roe} and T depth D_{Roe} of RA were derived as Eq. (13) and Eq. (14), respectively. First, we assumed that decomposing the Toffoli gate would result in Q of 11 and T depth of 3.

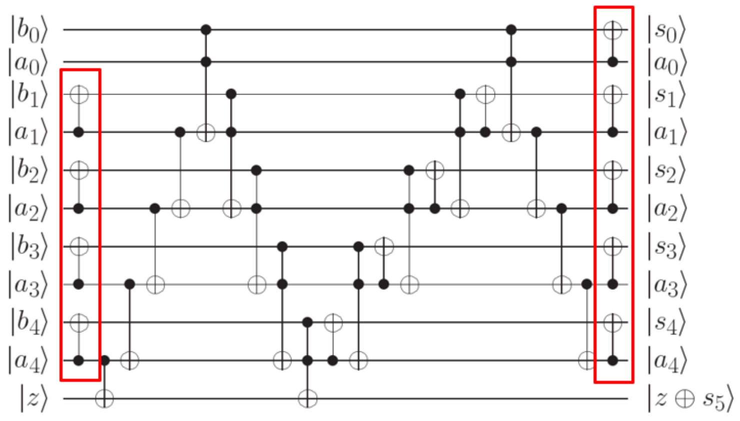

Figure 1. The circuit of Takahashi adder[1]. In order to avoid simultaneous $CNOT$ operation, we assumed that the $CNOT$ gates in the red box are performed serially.

Figure 1 shows the circuit of Takahashi adder. The RA uses the Takahashi adder to add the values of the two different quantum states. Although there are many $CNOT$ gates that can be performed simultaneously in the Takahashi adder, our analysis assumes that the $CNOT$ gates are not performed simultaneously to minimize the number of additional logical qubits for lattice-surgery-based $CNOT$ operations. The Takahashi adder serially performs about $5n$ $CNOT$ gates, $2n$ toffoli gates. Therefore, the elementary gate step $Q_{TA,serial}$ and T depth $D_{TA,serial}$ of the Takahashi adder are expressed as

$$Q_{TA,serial} = 27n,\ D_{TA,serial} = 6n. \tag{1}$$

The constant term was ignored as its influence was small. Häner's constant adder includes serial and parallel versions, and the RA uses a serial version of a constant adder.

Figure 2 shows the circuit of constant adder. Let us define $Q(A)(T(A))$ as the elementary gate step(T depth) of the A operation. Then the elementary gate step and T depth of the constant adder are expressed as

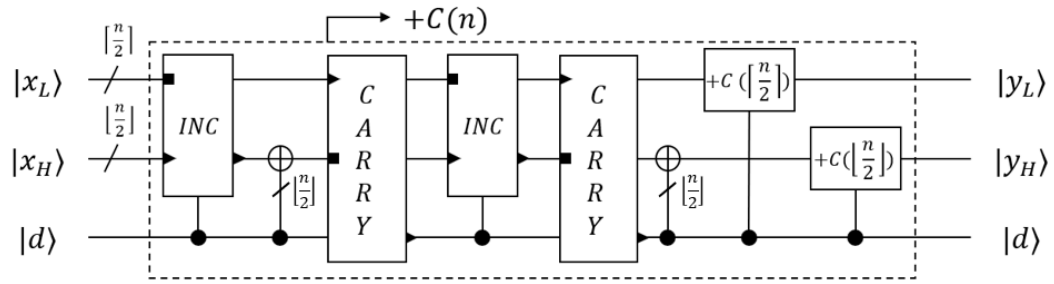

Figure 2. The circuit of constant adder. $C(n)$ represents n bit constant adder operation. INC represents incrementor operation. $CARRY$ represents carry operation. The black square represents the ancilla qubits used indirectly in the operation. The triangle inside the box represents the input of the operation, and the triangle outside the box represents the output of the operation.

$$
\begin{aligned}
Q(C(n)) &= Q(2\cdot INC(\lfloor \tfrac{n}{2} \rfloor)) + Q(2\cdot CARRY(\lfloor \tfrac{n}{2} \rfloor)) + 2\lfloor \tfrac{n}{2} \rfloor + Q(C(\lceil \tfrac{n}{2} \rceil)) + Q(C(\lfloor \tfrac{n}{2} \rfloor)) \\
&\approx Q(INC(n)) + Q(CARRY(n)) + n + Q(C(\lceil \tfrac{n}{2} \rceil)) + Q(C(\lfloor \tfrac{n}{2} \rfloor)),
\end{aligned} \tag{2}
$$

$$
\begin{aligned}
D(C(n)) &= D(2\cdot INC(\lfloor \tfrac{n}{2} \rfloor)) + D(2\cdot CARRY(\lfloor \tfrac{n}{2} \rfloor)) + D(C(\lceil \tfrac{n}{2} \rceil)) + D(C(\lfloor \tfrac{n}{2} \rfloor)) \\
&\approx D(INC(n)) + D(CARRY(n)) + D(C(\lceil \tfrac{n}{2} \rceil)) + D(C(\lfloor \tfrac{n}{2} \rfloor)).
\end{aligned} \tag{3}
$$

Therefore, it is necessary to know the $Q(INC(n))$, $D(INC(n))$, $Q(CARRY(n))$, and $D(CARRY(n))$ in order to obtain the elementary gate step and T depth of the constant adder. We used $CARRY$ suggested in Häner's work[2]. Assuming the worst case, the n bit $CARRY$ operation, including initialization of the dirty ancilla qubits, serially performs approximately $4n$ toffoli gates, $2n$ X gates, and $2n$ $CNOT$ gates. Therefore, $Q(CARRY(n)) = 48n$, $D(CARRY(n)) = 12n$. Similar to Haner's work, we used the incrementor proposed in[3]. The incrementor uses two Takahashi adder and $2n$ X gates. Because the X gate can be performed simultaneously, $Q(INC(n)) = 2\cdot Q_{TA,serial} = 54n$, $D(INC(n)) = 2\cdot D_{TA,serial} = 12n$. The elementary gate step and T depth of Controlled incrementor is the same as incrementor.

Therefore, the elementary gate step $Q_{CA,serial}$ and T depth $D_{CA,serial}$ of the constant adder gate are expressed as

$$
Q_{CA,serial} = 103n\log_2 n - 206n,\ D_{CA,serial} = 24n\log_2 n - 48n. \tag{4}
$$

A modular constant adder was constructed using two comparators and two constant adders. The comparator described in Häner's work was used. Therefore, the elementary gate step Q_{MCA} and T depth D_{MCA} of the modular adder are expressed as

$$
Q_{MCA} = 206n\log_2 n - 316n,\ D_{MCA} = 48n\log_2 n - 72n. \tag{5}
$$

A modular adder is constructed using one comparator, one Takahashi adder, and two constant adders. Therefore, the elementary gate step Q_{MA} and T depth D_{MA} of the modular adder are expressed as

$$
Q_{MA} = 206n\log_2 n - 337n,\ D_{MA} = 48n\log_2 n - 78n. \tag{6}
$$

In the controlled modular adder, only the Takahashi adder from the modular adder needs to be changed to a controlled version. Therefore, the elementary gate step Q_{CMA} and T depth D_{CMA} of the controlled modular adder are expressed as

$$
Q_{CMA} = 206n\log_2 n - 327n,\ D_{CMA} = 48n\log_2 n - 75n. \tag{7}
$$

Modular negation is constructed using one incrementor and a constant adder. The incrementor described in Häner's work was used. Therefore, the elementary gate step Q_{MN} and T depth D_{MN} of modular negation are expressed as

$$Q_{MN} = 103n\log_2 n - 152n,\ D_{MN} = 24n\log_2 n - 36n. \tag{8}$$

Modular multiplication is performed using n-controlled Takahashi adders and n modular constant adders. Therefore, the elementary gate step Q_{MM} and T depth D_{MM} of modular multiplication are expressed as

$$Q_{MM} = 206n^2\log_2 n - 279n^2,\ D_{MM} = 48n^2\log_2 n - 63n^2. \tag{9}$$

The modular square has the same number of elementary gate steps and T depth as modular multiplication.

The modular inversion in the RA uses a $4n$-controlled adder, $16n$-triple-controlled doubling, and $8n$-triple-controlled adder. As there are two logical qubits that maintain $|0\rangle$ while performing triple-controlled doubling and adders in the modular inversion circuit, we can easily implement triple-controlled operations using these $|0\rangle$ states. For example, *CCCCNOT* gate using two $|0\rangle$ states can be implemented as shown in Figure 3.

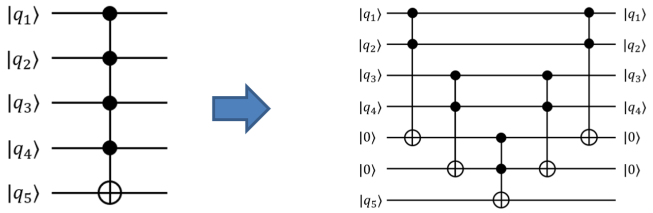

Figure 3. Implementation method of *CCCCNOT* gate using two $|0\rangle$ states.

Therefore, *CCCCNOT* gate using two $|0\rangle$ states uses five toffoli gates. The triple-controlled adder can be implemented as shown in Figure 4.

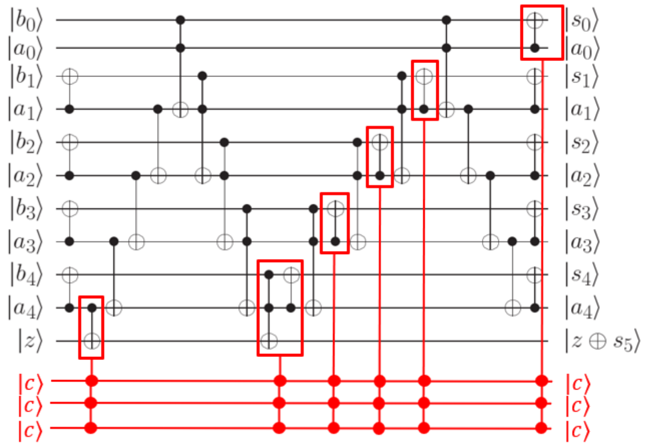

Figure 4. Implementation method of triple-controlled Takahashi adder.

Because the triple-controlled Takahashi adder uses $4n$ *CNOT* gates, $2n$ toffoli gates, n CCCCNOT gates, the elementary gate step and T depth of triple-controlled Takahashi adder are $4n + 11 \cdot (2n + 5 \cdot n) = 81n$ and $3 \cdot (2n + 5 \cdot n) = 21n$, respectively. Similarly, the elementary gate step and T depth of triple-controlled doubling are $2n + 11 \cdot 5 \cdot n = 57n$ and $3 \cdot 5 \cdot n = 15n$, respectively. The elementary gate step Q_{MI} and T depth D_{MI} of the modular inversion are expressed as

$$Q_{MI} = 1708n^2,\ D_{MI} = 444n^2. \tag{10}$$

Point addition in the RA uses four modular inversions, four modular multiplications, two modular squares, two modular constant adders, one modular negation, and one controlled modular adder. Therefore, the elementary gate step Q_P and T depth D_P of point addition are expressed as

$$Q_P = 1236n^2\log_2 n + 5158n^2 + 721n\log_2 n - 1111n, \tag{11}$$
$$D_P = 288n^2\log_2 n + 1398n^2 + 168n\log_2 n - 255n. \tag{12}$$

The RA uses $2n$ point addition. Therefore, the elementary gate step Q_{Roe} and T depth D_{Roe} of the RA are expressed as:

$$Q_{Roe} = 2472n^3\log_2 n + 10316n^3 + 1442n^2\log_2 n - 2222n^2, \tag{13}$$
$$D_{Roe} = 576n^3\log_2 n + 2796n^3 + 336n^2\log_2 n - 510n^2. \tag{14}$$

As the $CNOT$ gate is serially executed in the Takahashi adder, there are no cases where two or more $CNOT$s are executed simultaneously. Thus, $N_{CNOT} = 1$. In addition, because three T gates are used simultaneously in the Toffoli gate, and because there are no cases where the Toffoli gate is used simultaneously in the entire algorithm, the maximum number of T gates used simultaneously throughout the algorithm is $N_T = 3$.

Decomposition of parallelized constant adder RA at the logical level

We determine the elementary gate step and T depth of the RA when using the parallel constant adder. Throughout the analysis, the constant term was ignored because its influence was small. Unlike in the case of using the serial constant adder, the $CNOT$ gates of the Takahashi adder can be performed in parallel because b_n logical qubits have already been prepared to simultaneously perform the $CNOT$ gate. The elementary gate step $Q_{TA,parallel}$ and T depth $D_{TA,parallel}$ of the Takahashi adder are expressed as

$$Q_{TA,parallel} = 25n,\ D_{TA,parallel} = 6n. \tag{15}$$

Because the $CNOT$s in the Takahashi adder can be performed in parallel, $Q(INC_{parallel}(n)) = 50n$, $D(INC_{parallel}(n)) = 12n$. The elementary gate step and T depth of the parallelized constant adder are expressed as

$$\begin{aligned} Q(C_{parallel}(n)) &= Q(2\cdot INC_{parallel}(\lfloor\frac{n}{2}\rfloor)) + Q(2\cdot CARRY(\lfloor\frac{n}{2}\rfloor)) + 2\lfloor\frac{n}{2}\rfloor + Q(C_{parallel}(\lceil\frac{n}{2}\rceil)) \\ &\approx Q(INC_{parallel}(n)) + Q(CARRY(n)) + n + Q(C_{parallel}(\lceil\frac{n}{2}\rceil)), \end{aligned} \tag{16}$$

$$\begin{aligned} D(C_{parallel}(n)) &= D(2\cdot INC_{parallel}(\lfloor\frac{n}{2}\rfloor)) + D(2\cdot CARRY(\lfloor\frac{n}{2}\rfloor)) + D(C_{parallel}(\lceil\frac{n}{2}\rceil)) \\ &\approx D(INC_{parallel}(n)) + D(CARRY(n)) + D(C_{parallel}(\lceil\frac{n}{2}\rceil)). \end{aligned} \tag{17}$$

Therefore, the elementary gate step $Q_{CA,parallel}$ and T depth $D_{CA,parallel}$ of the parallel constant adder gate are expressed as

$$Q_{CA,parallel} = 198n,\ D_{CA,parallel} = 48n. \tag{18}$$

Using these modifications, we can redefine the elementary gate step and depth of the parallelized constant adder RA. The elementary gate step $Q_{Roe,parallel}$ and T depth $D_{Roe,parallel}$ of the parallelized constant adder RA are expressed as

$$Q_{Roe,parallel} = 19796n^3 + 3422n^2, \tag{19}$$
$$D_{Roe,parallel} = 5100n^3 + 834n^2. \tag{20}$$

Decomposition of Takahashi adder version RA at the logical level

We determine the elementary gate step and T depth of the Takahashi addder version RA. As in the RA, it is assumed that the $CNOT$ gate is serially performed on Takahashi adder to minimize N_{CNOT}. The modular p adder in the Takahashi adder version RA is constructed using one comparator and three Takahashi adders. Therefore, the elementary gate step $Q_{MA,p}$ and T depth $D_{MA,p}$ of the modular p adder are expressed as

$$Q_{MA,p} = 139n,\ D_{MA,T} = 33n. \tag{21}$$

In the controlled modular p adder, only the first Takahashi adder from the modular adder must be changed to a controlled version. Therefore, the elementary gate step $Q_{CMA,p}$ and T depth $D_{CMA,p}$ of the controlled modular p adder are expressed as

$$Q_{CMA,p} = 149n,\ D_{CMA,p} = 36n. \tag{22}$$

The modular p negation in Takahashi adder version was constructed using one incrementor and one Takahashi adder. Therefore, the elementary gate step $Q_{MN,p}$ and T depth $D_{MN,p}$ of modular p negation are expressed as

$$Q_{MN,p} = 81n,\ D_{MN,p} = 18n. \tag{23}$$

Modular p multiplication is constructed using n-controlled Takahashi adders and n modular adders. Therefore, the elementary gate step $Q_{MM,p}$ and T depth $D_{MM,p}$ of modular p multiplication are expressed as

$$Q_{MM,p} = 176n^2,\ D_{MM} = 42n^2. \tag{24}$$

The modular p square has the same number of elementary gate steps and T depth as modular multiplication.

The point addition in the RA uses 4 modular inversion, 4 modular p multiplication, 2 modular p square, 2 steps of the modular constant adder, 1 modular p negation, and 1 controlled modular p adder. Therefore, the elementary gate step $Q_{P,T}$ and T depth $D_{P,T}$ of point addition are expressed as

$$Q_{P,T} = 7888n^2 + 412n\log_2 n - 402n, \tag{25}$$
$$D_{P,T} = 2028n^2 + 96n\log_2 n - 90n. \tag{26}$$

Because the RA uses a $2n$ point addition, the elementary gate step $Q_{Roe,T}$ and T depth $D_{Roe,T}$ of the Takahashi adder version RA are expressed as

$$Q_{Roe,T} = 15776^3 + 824n^2\log_2 n - 804n^2, \tag{27}$$
$$D_{Roe,T} = 4056n^3 + 192n^2\log_2 n - 180n^2. \tag{28}$$

Because Takahashi adder is performed serially, as in the RA, $N_{CNOT} = 1$ and $N_T = 3$.

Comparison of physical qubit number and required time between RA using Takahashi adder and RA using CDKM adder

Figure 5 shows the circuit of CDKM adder. The CDKM adder serially performs about $3n$ $CNOT$ gates, $2n$ toffoli gates. Therefore, the elementary gate step Q_{CDKM} and T depth D_{CDKM} of the Takahashi adder are expressed as

$$Q_{CDKM} = 25n,\ D_{CDKM} = 6n. \tag{29}$$

Like Takahashi adder version RA, only the constant adder used in the modular p operation are replaced with the CDKM adder. Therefore, the elementary gate step $Q_{Roe,CDKM}$ and T depth $D_{Roe,CDKM}$ of the CDKM adder version RA are expressed as

$$Q_{Roe,CDKM} = 15660^3 + 792n^2\log_2 n - 760n^2, \tag{30}$$
$$D_{Roe,CDKM} = 4056n^3 + 192n^2\log_2 n - 180n^2. \tag{31}$$

CDKM adder has an advantage in elementary gate step compared to Takahashi adder, but has the disadvantage of requiring the use of one additional logical ancilla qubit. Additionally, since T depth, which has a significant impact on the time required, is the same when using the Takahashi adder and when using the CDKM adder, there is no significant benefit in terms of time.

Table 1 shows the N_{phy} and required time of Takahashi adder verison RA and CDKM adder version RA. We assumed $\varepsilon_p = 10^{-3}$, $c_t = 200ns$, and $p_{fail} = 0.01$. Although there may actually be an error because only the time required for the T gate was considered when calculating the time required, Takahashi adder version RA and CDKM adder version RA take the same time. Additionally, CDKM adder version RA uses slightly more physical qubits than Takahashi adder version RA. Therefore, we conducted the analysis in consideration of Takahashi adder, which is the most efficient among several adders.

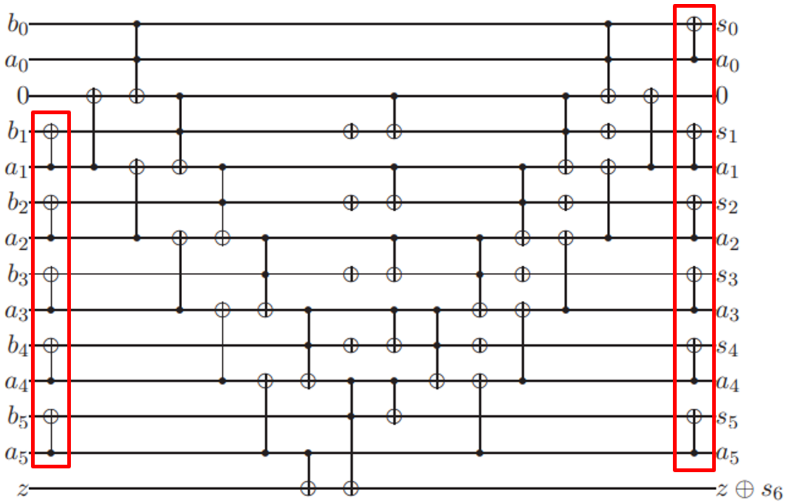

Figure 5. The circuit of CDKM adder[4]. In order to avoid simultaneous *CNOT* operation, we assumed that the *CNOT* gates in the red box are performed serially.

Table 1. Comparing number of physical qubits and required time of Takahashi adder version RA with CDKM adder version RA. $T_{RA,Tak}(N_{RA,Tak})$ represents required time(number of physical qubits) of Takahashi adder version RA and $T_{RA,CDKM}(N_{RA,CDKM})$ represents required time(number of physical qubits) of CDKM adder version RA.

bit length	160	192	224	256	384	521
$T_{RA,Tak}(s)$	8.52×10^5	1.40×10^6	2.22×10^6	3.31×10^6	1.18×10^7	3.09×10^7
$T_{RA,CDKM}(s)$	8.52×10^5	1.40×10^6	2.22×10^6	3.31×10^6	1.18×10^7	3.09×10^7
$N_{RA,Tak}$	3.762×10^6	4.446×10^6	5.061×10^6	5.676×10^6	9.158×10^6	1.360×10^7
$N_{RA,CDKM}$	3.764×10^6	4.448×10^6	5.063×10^6	5.677×10^6	9.160×10^6	1.360×10^7

References

1. Takahashi, Y., Tani, S. & Kunihiro, N. Quantum addition circuits and unbounded fan-out. *Quantum Inf. & Comput.* **10**, 872–890 (2010).
2. Häner, T., Roetteler, M. & Svore, K. M. Factoring using 2n+ 2 qubits with toffoli based modular multiplication. *Quantum Inf. & Comput.* **17**, 673–684 (2017).
3. Gidney, C. Stackexchange: Creating bigger controlled nots from single qubit, toffoli, and cnot gates, without workspace. 2015. *URL: http://cs. stackexchange. com/questions/40933/creating-biggercontrolled-nots-from-single-qubit-toffoli-and-cnot-gateswith* .
4. Cuccaro, S. A., Draper, T. G., Kutin, S. A. & Moulton, D. P. A new quantum ripple-carry addition circuit. *arXiv preprint quant-ph/0410184* (2004).
